# Tip-Variant Focal Segmental Glomerulosclerosis in a Patient With Primary Hypothyroidism: A Case Report

**DOI:** 10.7759/cureus.30020

**Published:** 2022-10-07

**Authors:** Jayashankar CA, Pavan LR, Mohammed Ishaq, Pruthvi R S, Amey Joshi

**Affiliations:** 1 Internal Medicine, Vydehi Institute of Medical Sciences and Research Centre, Bangalore, IND; 2 General Medicine, Vydehi Institute of Medical Sciences and Research Centre, Bangalore, IND

**Keywords:** kidney disease, focal segmental glomerulosclerosis (fsgs), nephrotic syndrome, primary hypothyroidism, tip-variant

## Abstract

Focal segmental glomerulosclerosis (FSGS) is a common cause of nephrotic syndrome (NS) and one of the leading causes of end-stage kidney disease. Endocrinological abnormalities due to the urinary loss of hormone-binding proteins, such as transient hypothyroidism, are well documented in FSGS. Secondary FSGS can arise due to viral infections, drugs, and pre-existing glomerular diseases. Few reports have highlighted the occurrence of FSGS in the background of hypothyroidism. We present a case of a young male with primary hypothyroidism who developed the tip variant of FSGS. A combination of oral corticosteroids and angiotensin-converting enzyme (ACE) inhibitors was successful in causing remission of the FSGS with no relapse.

## Introduction

The presence of partial tuft sclerosis in some glomeruli from a renal biopsy specimen is referred to as focal segmental glomerulosclerosis (FSGS). FSGS is broadly classified into primary and secondary forms, with the latter caused by an underlying etiology and, more often, accompanied by an increased occurrence of endocrinological disorders [1]. Although transient hypothyroidism in FSGS has been well-documented to occur due to urinary protein losses, literature on the occurrence of FSGS in the background of primary hypothyroidism is limited [2-5]. We present a rare case of a young patient with a history of primary hypothyroidism who developed tip-variant FSGS.

## Case presentation

A 17-year-old male presented to our tertiary care hospital with complaints of generalized edema involving the legs, thighs, scrotum, and abdomen with periorbital and facial puffiness, which was insidious in onset and progressed over six weeks, accompanied by frothy urine without haematuria or reduction in urine output. His medical history was significant for primary hypothyroidism; he was on treatment with levothyroxine supplementation for one year. There was no history of fever, skin rashes, joint pain, or other acute or chronic drug use. On examination, bilateral pitting pedal edema, facial puffiness, and scrotal and penile edema were observed. In addition, reduced breath sounds were noted in both lung bases. Gastrointestinal, neurological, and cardiovascular system examinations were unremarkable.

Laboratory investigations were significant for hypoalbuminemia, hypercholesterolemia, high spot urine protein/creatinine ratio, and elevated erythrocyte sedimentation rate. Antinuclear antibody titers and complement levels (C3, C4) were within normal limits. Viral markers of human immunodeficiency virus (HIV), hepatitis B virus (HBV), and hepatitis C virus (HCV) enzyme-linked immunosorbent assay (ELISA) were negative (Table 1). Ultrasonography of the abdomen and pelvis revealed mild ascites with increased echotexture of both kidneys. Two-dimensional echocardiography showed normal left and right ventricular systolic function, an ejection fraction of 58%, and grade 1 left ventricular diastolic dysfunction. The patient had no other signs and symptoms suggestive of connective tissue disorders. 

**Table 1:** Laboratory investigations HDL: High-density lipoprotein, LDL: Low-density lipoprotein, VLDL: Very low-density lipoprotein, HIV: Human immunodeficiency virus, HBV: Hepatitis B virus, HCV: Hepatitis C virus.

Laboratory Investigations:	At time of admission:	Normal Values:
White blood count (cells/cumm)	14,900	4,000-11,000
Hemoglobin (g/dl)	13.6	13.0-17.0
Platelet count (lakhs/cumm)	3.45	1.5-4.1
Peripheral smear	Normocytic normochromic blood picture	Normocytic normochromic blood picture
Erythrocyte sedimentation rate (mm/hr.)	53	0-10
Serum Creatinine (mg/dl)	1.08	0.5-1.10
Blood Urea (mg/dl)	36.38	11-40
Total Bilirubin (mg/dl)	0.3	0.3-1.2
Direct Bilirubin (mg/dl)	0.25	0.0-0.2
Total Protein (g/dl)	3.2	6.0-8.0
Albumin (g/dl)	<1	3.5-5.2
Globulin (g/dl)	2.2	1.8-3.6
Aspartate aminotransferase (IU/L)	23	15-40
Alanine aminotransferase (IU/L)	15	10-40
Alkaline phosphatase (IU/L)	126	53-128
Prothrombin time (seconds)	12.9	10.02-2.98
International Normalized Ratio	1.12	0.8-1.2
Partial thromboplastin time (seconds)	35	22-37
Urine Nitrite	Negative	Negative
Urine Leucocytes	Nil	Nil
Urine Albumin (mg/dl)	>1000	<30
Urine Glucose (mg/dl)	Nil	Nil
Urine Blood (Hemoglobin) (mg/dl)	0.03	<0.03
Urine Epithelial cells	2-3” HPF	1-4” HPF
Urine Erythrocytes	5-6” HPF	0-4” HPF
Urine Casts	Nil	Nil
Urine Pus cells	2-3” HPF	Nil
C-reactive protein (mg/dl)	1.14	<0.8
Anti-nuclear antibody	1+ (Homogenous)	Negative
Complement level C3 (mg/dl)	108	79-152
Complement level C4 (mg/dl)	27.6	16-38
Thyroid stimulating hormone (mIU/ml)	3.60	0.4-4.2
Free T3 (pg/ml)	2.97	2.3-4.1
Free T4 (mg/ml)	1.3	0.9-1.7
Anti-Thyroid peroxidase antibodies (IU/ml)	0.3	0-9
Spot urine protein/creatinine ratio (mg/mg)	7.29	<0.2
Serum Sodium (mEq/L)	135	136-145
Serum Potassium (mEq/L)	2.96	3.5-5.1
Serum Chloride (mEq/L)	109	98-107
Serum Calcium (mg/dl)	7.1	8.4-10.2
Random plasma glucose (mg/dl)	128	100-140
Total Cholesterol (mg/dl)	570	150-200
Triglycerides (mg/dl)	206	<150
HDL Cholesterol (mg/dl)	46.5	40-65
LDL Cholesterol (mg/dl)	442	<100
VLDL (mg/dl)	41.2	2-30
Serology (HIV, HBV, HCV) ELISA	Negative	Negative
Automated Blood culture and sensitivity	Negative	Negative
SARS CoV-2 RTPCR	Negative	Negative

**Figure 1:**
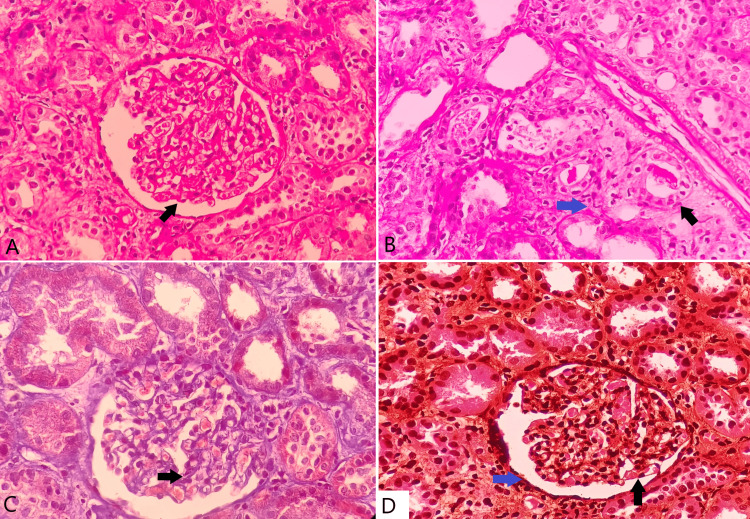
Histopathology slides of renal biopsy specimen A) H&E section with black arrow showing glomerulus with segmental sclerosis at the tubular pole. B) H&E section with black arrow showing tubule containing granular cast and blue arrow showing interstitium containing infiltrate composed of polymorphs, lymphocytes, and plasma cells. C) Masson trichome stained section with arrow showing sclerosis and collagen deposition in the glomerulus. D) Jones methenamine silver-stained section with black arrow showing normal basement membrane of glomerular capillary and blue arrow showing normal basement membrane of Bowman’s capsule.

Based on clinical and biochemical parameters suggestive of nephrotic syndrome (NS), an ultrasound-guided renal biopsy was performed, which revealed FSGS involving 3/10 glomeruli showing segmental sclerosis at the tubular pole with no mesangial or endocapillary hypercellularity, crescent, or necrosis. Tubules contained a single granular cast and occasional uromodulin casts. The interstitium contained a mixed infiltrate composed of polymorphs, lymphocytes, and plasma cells with no features of active tubulitis (Figure 1). 

The patient was commenced on oral prednisolone for four weeks and angiotensin-converting enzyme (ACE) inhibitors and statins. The patient was followed up telephonically and continued to be in remission with a gradual improvement in serum albumin and a decrease in proteinuria. The patient is symptom-free after six months of follow-up.

## Discussion

FSGS is a common cause of NS, accounting for 40% of cases in adults and 20% in children, and is a leading cause of end-stage renal disease (ESRD) in over 50% of patients affected by it [6,7]. Transient hypothyroidism due to urinary loss of thyroid binding globulin and bound thyroxine has been well-documented and often warrants thyroid function assessment in patients with NS [2]. However, few cases of FSGS have been reported in patients as a consequence of hypothyroidism [3-5]. We describe a young male with primary hypothyroidism presenting with FSGS. 

FSGS can be categorized into presumed permeability factor-related FSGS (ppfFSGS), traditionally known as primary FSGS, secondary FSGS, genetic FSGS, and FSGS of undetermined cause. PpfFSGS is thought to be caused by circulating permeability factors that cause injury to podocytes, including anti-CD40 antibody, apolipoprotein A1, calcium/calmodulin-serine protein kinase (CASK), cardiotrophin-like cytokine factor 1 (CLCF-1), and urokinase-type plasminogen activator receptor (utPAR). Immunosuppressive therapies like glucocorticoids and calcineurin inhibitors have been found to induce remission in nearly 70% of ppfFSGS with little to no role of renin-angiotensin system (RAS) inhibitors. Secondary FSGS can be classified into maladaptive FSGS, drug-induced FSGS (due to bisphosphonates, interferons, heroin, analgesics, lithium, and androgens), viral-induced FSGS (due to hepatitis B and C, HIV, parvovirus B19, cytomegalovirus (CMV), and SARS-CoV-2), and superimposed FSGS on pre-existing glomerular disease. In maladaptive FSGS, where podocyte damage is due to abnormal hemodynamic stress, treatment is aimed at reducing glomerular capillary hypertension using RAS inhibitors. Genetic FSGS has been commonly observed in patients with mutations in structural glioblastoma multiforme (GBM) glycoproteins, including mutations in COL4, EMP2, WT1, NPHS1, and APOL1 genes. FSGS of undetermined significance is a category of FSGS with features similar to that of secondary FSGS with no identifiable cause [8]. The common causes of secondary FSGS in the present case were excluded, i.e., infectious and drug-induced etiologies. Hypothyroidism can alter kidney function by its effect on cardiac output, RAS, decreased truncated renal tubular mass, and altered glomerular structure [9]. However, literature supporting this conjecture is limited, and studies exploring the possible linkage may be helpful. Patients with hypothyroidism and NS have also been observed to have higher urinary excretion of protein than those with normal thyroid function, which can further compound the complications associated with NS [2]. 

The spectrum of FSGS encompasses disease entities characterized by podocyte injury with foot process effacement leading to the histological pattern of obliteration of glomerular capillaries by extracellular matrix (ECM) accumulation [10]. FSGS is characterized by the presence of sclerosis in parts (segmental) of at least one glomerulus (focal) in the entire kidney biopsy specimen when examined by light microscopy (LM), immunofluorescence (IF), or electron microscopy (EM). In 2004, the Columbia classification was proposed for the pathological evaluation of FSGS [11]. The following are the five FSGS variants: collapsing, cellular, glomerular tip, peri-hilar, and not otherwise specified (NOS). NOS was the most common pathological variant (62.2%), followed by peri-hilar (11.2%), cellular (9.4%), glomerular tip (7.7%), and collapsing (4.3%) [3]. FSGS tip variant is defined, according to the Columbia classification of FSGS, as "the presence of at least one segmental lesion involving the tip domain (i.e., the outer 25% of the tuft next to the origin of the proximal tubule) with either adhesion between the tuft and Bowman's capsule at the tubular lumen or neck, or confluence of podocytes with parietal or tubular epithelial cells at the tubular lumen or neck" [11]. In the present case, FSGS of the tip variant was identified, which responded well to a course of steroids. However, as the cause of the FSGS could not be identified, ACE inhibitors were also commenced to reduce intra-renal capillary pressures and proteinuria. Renal biopsy can also often help differentiate between primary FSGS and maladaptive FSGS. EM in primary FSGS and maladaptive FSGS demonstrates diffuse foot process effacement (FPE) (>80%) and segmental FPE, respectively. On LM, maladaptive FSGS can show perihilar sclerosis, signs of scarring, FGGS in multiple glomeruli, and glomerulomegaly, all of which are not frequently seen in primary FSGS [12]. Determining the type and variant also helps guide therapeutic intervention strategies and gauging prognosis and long-term renal outcomes. 

Steroids and immunomodulatory therapies serve as the mainstay of FSGS treatment. These agents have established genomic and non-genomic immunomodulatory properties, including suppressing humoral and cellular immunity. Glucocorticoids may also promote podocyte survival and actin cytoskeleton stabilization, increasing the resistance to injury [13]. Several reports in nephrotic patients with glomerular tip lesion (GTL) suggest an excellent response to steroids and a favourable course like that of minimal-change disease rather than FSGS. However, the natural course of primary FSGS unresponsive to treatment is frequently relentless, with 50% of patients progressing to ESRD within 3-8 years from diagnosis [14]. These patients are more prone to infections, thrombosis, and cardiovascular disease, which can lead to mortality [15]. In the present case, the patient had an excellent response to a combination of oral steroids and ACE inhibitors with gradual improvement in symptoms and hemodynamic parameters. Although a worsening of his thyroid status was expected due to the current episode of NS, levothyroxine was continued at the initial dosage with close monitoring of his thyroid function. However, we suggest that thyroid status be actively monitored during NS presentations with appropriate dose adjustments. 

## Conclusions

The present case highlights the occurrence of FSGS in a young male with a background of hypothyroidism. Longitudinal studies analyzing kidney disease in primary hypothyroidism may provide evidence regarding the association between the two. The tip variant of FSGS, as seen in our case, has often been described to have a favorable prognosis with high steroid response rates. In patients with hypothyroidism with FSGS, it is crucial to monitor thyroid function and be watchful for signs of worsening hypothyroidism due to the massive urinary loss of proteins and the increased risk of complications, including thrombosis and infection.
